# Equilibrium state dynamics‐based modeling of temporal dose delivery dependencies of FLASH skin sparing

**DOI:** 10.1002/mp.70143

**Published:** 2025-11-21

**Authors:** Till Tobias Böhlen, Veljko Grilj, Anouk Sesink, Preethi Devanand, Brita Singers Sørensen, Per Poulsen, Luca Soutter, Jean‐François Germond, Claude Bailat, François Bochud, Fernanda G. Herrera, Raphaël Moeckli

**Affiliations:** ^1^ Institute of Radiation Physics Lausanne University Hospital and Lausanne University Lausanne Switzerland; ^2^ Department of Radiation Oncology Lausanne University Hospital and Lausanne University Lausanne Switzerland; ^3^ Danish Centre for Particle Therapy Aarhus University Hospital Aarhus Denmark

**Keywords:** FLASH, mice skin toxicity, modeling, ultra‐high dose rate (UHDR)

## Abstract

**Background:**

FLASH radiotherapy shows promise in sparing normal tissues while maintaining tumor control, with the magnitude of its sparing effect being strongly influenced by the temporal dose delivery (TDD) structure of ultra‐high dose rate (UHDR) irradiation. Quantitatively describing these dependencies is critical for optimizing UHDR treatments and guiding preclinical and clinical applications.

**Purpose:**

This study introduces perturbed equilibrium state dynamics (ESD) as a minimal phenomenological framework to describe TDD dependencies of FLASH normal tissue sparing.

**Methods:**

ESD‐based modeling assumes a transient perturbation of a generic equilibrium state during irradiation, modulating the biological system's instantaneous radiosensitivity. Isoeffective dose ratios (FLASH‐modifying factors, FMF) were derived from a collection of acute skin toxicity data, consisting of both previously published and newly acquired data, from 721 mice across 60 experimental groups irradiated with pulsed electron beams and scanned proton beams spanning various TDD patterns. Eight ESD model variants were evaluated based on their ability to reproduce experimental FMF values, optimizing their three or four free parameters and assessing goodness‐of‐fit metrics.

**Results:**

The best‐ranked model based on the Bayesian Information Criterion (BIC) used three parameters to reproduce accurately 52 averaged FMF values (mean absolute error: 0.034, mean absolute percentage error: 4.3%). It consistently reproduces experimentally observed features of FLASH sparing in terms of FMF, including an increased sparing with dose followed by saturation at high doses. Two additional model variants received similar support by BIC, while the remaining five variants were less favored.

**Conclusions:**

ESD‐based modeling offers an effective framework to describe TDD dependencies of FLASH murine skin sparing while limiting complexity and number of free parameters to avoid overfitting. Through parameter tuning, the ESD modeling aligns with key experimental data, supporting its potential as a predictive, parsimonious framework for experimental and translational research. By highlighting the potential role of a transient equilibrium perturbation modulating instantaneous radiosensitivity and quantifying respective measures, ESD‐based models may also guide the exploration and modeling of underlying mechanisms. While the present application is tissue‐specific, the approach is broadly adaptable, with clinical translation requiring refitting and validation in other contexts.

## 1 INTRODUCTION

Experimental evidence suggests that the normal tissue‐sparing effect of FLASH radiotherapy (FLASH‐RT) may be influenced by temporal dose delivery parameters, including average dose rate, exposure duration, beam pulse structure, and delivery pauses.^1^, ^2^, ^3^, ^4^, ^5^, ^6^ For preclinical research and clinical translation, it is therefore essential to understand these dependencies to optimize the design of ultra‐high dose rate (UHDR) devices and treatment approaches, as well as to guide future experimental and translational research.

The modeling of the FLASH effect is currently hampered by a limited, elusive understanding of its underlying mechanisms ^5^, ^7^, ^8^, ^9^ and, from a modeling perspective, by its scarce experimental characterization. Mechanistic, process‐oriented models of the FLASH effect risk being overly complex and tend to obscure the essential temporal role of the irradiations, reducing their practical applicability and requiring extensive parameter tuning. Oxygen enhancement ratio (OER)‐based modeling has been shown to capture temporal dose delivery dependencies,^10^, ^11^ but is difficult to reconcile with oxygen depletion measurements and modeling.^12^, ^13^, ^14^

In this study, we propose a phenomenological modeling approach based on perturbed equilibrium state dynamics (ESD), where a biological system's instantaneous radiosensitivity is modulated by a transient perturbation of a generic equilibrium state. This modeling approach focuses on capturing experimentally observed effect behaviors in terms of isoeffective dose ratios as a function of the temporal dose delivery profiles and integrates well into current dose delivery frameworks without invoking complex biochemical pathways. It should be understood as a generalization of existing dose‐modifying approaches, rather than as an entirely new modeling approach. Its mathematical structure is inspired by dynamic equilibrium models commonly used in biology and ecology, where a latent quantity is transiently saturated or depleted and recovers toward equilibrium.^11^, ^15^, ^16^ We validate the model against an extensive dataset of acute murine skin reactions, drawing on both published studies and new in vivo measurements. This data‐driven methodology provides a simplified yet flexible foundation for quantifying temporal dose delivery dependencies of FLASH sparing, which are crucial for the clinical implementation of the FLASH effect and its further preclinical investigation.

## 2 METHODS

### 2.1 In vivo experimentation

Experiments were conducted in compliance with the guidelines established by the Animal Ethics Committee of Vaud, Switzerland, and were approved by license nos. VD3934 and VD3962 (DGAV Canton Vaud). Mice used within this research were acclimatized for 1 week prior to experimentation. Mice were maintained and handled in pathogen‐free conditions in cages with a maximum of five mice per cage, under a controlled 12 h light/dark cycle, relative humidity of 55%, and controlled temperature of 21°C, with food and sterile water provided daily, all in accordance with the guidelines provided by the institution. The experiments were conducted on female BALB/c mice (Charles River; France) at 8 weeks of age. The dorsal skin of 8‐week‐female old BALB/c mice was placed under ketamine anesthesia (75 µg/g body weight), supplemented with medetomidine (0.5 µg/g body weight) using intraperitoneal (IP) injection and followed by atipamezole (1 µg/g body weight) IP injection post‐irradiation.

Electron irradiations were conducted at our institute using the 5–6 MeV pulsed electron beam from the prototype linear accelerator Oriatron eRT6.^17^ The dorsal skin was gently lifted and secured to the back of a 3‐cm‐thick carbon applicator, featuring a square aperture measuring 15 mm × 15 mm, covered by a 0.5 mm thick PMMA layer. Only the portion of skin extending up to 10 mm mark behind the aperture was exposed to either UHDR or CDR treatments, while the remainder of the animal was shielded by the applicator. Considering that the extended skin was folded, the total irradiated area was 20 mm × 15 mm. A summary of key setup and irradiation parameters for electron skin irradiations for the two radiation modalities is provided in Table S1, following recommendations.^18^ Absolute dose measurements were performed using EBT‐XD GafChromic films, positioned identically to the extended mouse skin. Given that the folded mouse skin remained thinner than 1 mm, the dose measured by the film served as an accurate proxy for the dose absorbed by the skin layers. Skin reactions were monitored for 50 days post‐irradiation unless euthanasia was required earlier due to mice reaching severity endpoint of >3 mm skin ulceration, occurring 16–30 days post CDR‐ and UHDR‐irradiation. Normal tissue complication probability (NTCP) is expressed as the fraction of animals in a group that developed skin ulcers exceeding 3 mm in any direction. This criterion was established as the humane endpoint in compliance with the approved animal license.

### 2.2 Mouse skin toxicity data for model assessment

A collection of acute mouse skin toxicity data was used to assess the models’ capability of describing FLASH normal tissue sparing for different temporal dose delivery patterns. The data collection evaluated skin toxicity scores of 721 mice divided into 60 experimental groups irradiated either with a pulsed electron beam (see previous section) or with the entrance dose plateau of a scanned proton beam of 244–250 MeV and included dose‐response curves for conventional dose rate (CDR) and ultra‐high dose rate (UHDR) irradiations for both electron and proton beams. The proton experiments have been described in detail elsewhere^2^, ^19^ and currently represent the largest consistently acquired data series evaluating acute murine skin reactions after CDR and UHDR irradiation. A summary of the datasets used for model assessment is given in Table 1. They comprise diverse temporal dose delivery structures spanning a dose range of 18–53 Gy, time‐averaged dose rates (TADR) between 0.11 and 111 Gy/s, and exposure times of 0.21 s to 10 min, including dose rate scans and split‐beam deliveries.

**TABLE 1:** Summary of acute skin toxicity data used for model assessment.

Reference	Dataset ID	Label	Skin toxicity	Evaluation time	Mice groups	Mice total	Delivery type	Nominal energy (MeV)	Source	Total dose (Gy)	TADR (Gy/s)	Exposure time^a)^ (s)
This work	e1	CDR	Ulcers (>3 mm)	Max over 1–50 d	6	60	Pulsed broad electron beam (collimated)	5	Oriatron eRT6, PMB	18–31.9	0.11	165–292
This work	e2	UHDR	Ulcers (>3 mm)	Max over 1–50 d	7	70	Pulsed broad electron beam (collimated)	6	Oriatron eRT6, PMB	23.1–37	109–111	0.21–0.34
Sør22^19^	p1	CDR	Grade 1.5–3.5	Max over 11–25 d	11	154	Scanned proton pencil beam	244	ProBeam, Varian	23.2–39.2	0.35–0.40	61–107
Sør22^19^	p2	UHDR	Grade 1.5–3.5	Max over 11–25 d	14	147	Scanned proton pencil beam	250	ProBeam, Varian	31.4–52.8	65–92	0.35–0.73
Sør24^2^	p3	Varying DR no repaint	Grade 1.5–3.5	Max over 11–25 d	7	109	Scanned proton pencil beam	250	ProBeam, Varian	39.3	0.7–80	0.5–60.5
Sør24^2^	p4	Varying DR with repaint	Grade 1.5–3.5	Max over 11–25 d	8	86	Scanned proton pencil beam	250	ProBeam, Varian	39.3	0.37–80	0.44–107.8
Sør24^2^	p5	Total dose split into 1–6 equal deliveries with 2 min pauses	Grade 1.5–3.5	Max over 11–25 d	7	125	Scanned proton pencil beam	244–250	ProBeam, Varian	39.3	0.065–60	0.68–607.3

Abbreviations: CDR, Conventional dose rate; DR, dose rate; TADR, time‐averaged dose rate (from beginning to end of irradiation, including pauses when splitting the dose); UHDR, Ultra‐high dose rate.

^a)^ From beginning to end of irradiation, including pauses when splitting the dose.

### 2.3 Derivation of experimental isoeffective dose ratios

The FLASH‐modifying factor (FMF) is the ratio of doses required to be delivered at a *reference* temporal dose delivery pattern ($D_{\mathrm{ref}}$, i.e. a CDR irradiation) and at a *test* temporal dose delivery pattern ($D_{\mathrm{test}}$, i.e. any temporal irradiation pattern) to achieve an isoeffect for a given biological endpoint^1^

(1) $$\mathrm{FMF}={\left.\frac{D_{\mathrm{ref}}}{D_{\mathrm{test}}}\right|}_{\mathrm{isoeffect}}$$

**FIGURE 1:**
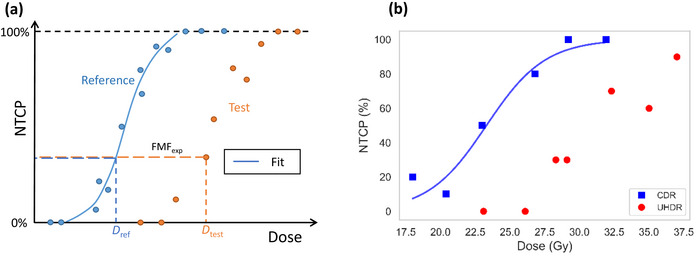
(a) Schematic representation: experimental dose ratios for isoeffective normal tissue complication probability (NTCP), referred to as experimental FLASH modifying factors (FMF_exp_), were computed utilizing the experimental data point for the test temporal dose delivery pattern (𝐷_test_) and the reference dose (𝐷_ref_) obtained from a logistic fit to the dose‐response curve for the respective reference temporal dose delivery pattern. (b) NTCP for skin ulcers (>3 mm) after conventional dose rate (CDR) and ultra‐high dose rate (UHDR) electron irradiation (i.e., datasets e1 and e2). A logistic NTCP curve (line) was fitted to the experimental single mouse data for CDR data.

This follows a definition analogous to the one of relative biological effectiveness for different radiation qualities.^20^ Experimental data provided group‐wise NTCP. Dataset e1 (TADR of 0.11 Gy/s) and dataset p1 (TADR of 0.35‐0.40 Gy/s), see Table 1, were used to establish reference dose‐response curves for electron and proton irradiations, respectively. For this purpose, a logistic NTCP curve was fitted to the experimental single mouse data for each evaluated skin toxicity grade. The experimental isoeffective dose ratios (FMF_exp_) were then calculated, as illustrated in Figure 1a, using the experimental data points to determine $D_{\mathrm{test}}$ and obtaining the corresponding $D_{\mathrm{ref}}$ for an isoeffective NTCP from the logistic fit to the respective reference dataset. Since isoeffective dose ratios are undefined for cases of complete absence or saturation of effect, FMF_exp_ was only determined for data points with an NTCP between 1% and 99%. For proton data, multiple grades of skin reactions were evaluated and resulted often in multiple FMF_exp_ values pertaining to different toxicity grades for the same mouse group. These FMF_exp_ values were averaged for model evaluation.

### 2.4 Equilibrium state dynamics‐based modeling and simulation of experimental isoeffective dose ratios

**FIGURE 2:**
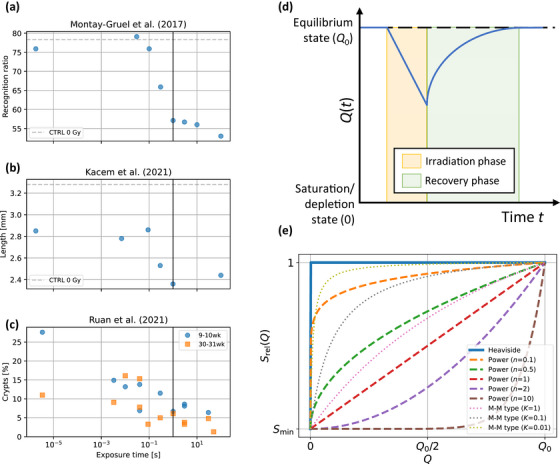
(a–c) Changes in normal tissue sparing for a fixed single fraction dose ((a) 10 Gy,^26^ (b) 10 Gy,^25^ (c) 11.2 Gy (9–10 weeks)/12.5 Gy (30–31 weeks)^4^) as a function of exposure time for different in vivo assays. Data points were digitized from the original publications.^4^, ^25^, ^26^ Dashed grey lines (CTRL) correspond to reference level for control groups. (d) Schematic graph illustrating perturbed equilibrium state dynamics of a generic quantity 𝑄(𝑡) that may be pushed away from its equilibrium state 𝑄_0_ by an irradiation at instantaneous dose rate $\dot{D}\left(t\right)$ inducing a change Δ(𝑄). 𝑄(𝑡) returns toward equilibrium 𝑄_0_ at a given recovery rate $\dot{R}\left(Q\right)$. (e) Evaluated dependencies for the instantaneous relative radiosensitivity Srel(𝑄) include a Heaviside function, a power function, and a Michaelis–Menten (M–M) type behavior. For details, see text and Table 2.

A recent meta‐analysis examining in vivo data on NT sparing by FLASH as a function of dose found that available experimental data can be described by a piecewise linear function of two parts when expressed as FMF‐weighted dose versus dose, also referred to as sudden effect transition (SET) function.^1^ This implies for UHDR irradiations a decreasing sparing effect in terms of FMF for lower doses and a saturation for very high doses. These features have been subsequently confirmed for data acquired over a large dose range,^21^, ^22^, ^23^ see Figure S1. Such a behavior is compatible with an instantaneous relative radiosensitivity $S_{\mathrm{rel}}$ that is initially one (compared to a low dose rate irradiation) and that may reach a minimum value $S_{\min}$ during UHDR irradiation. Furthermore, experiments have shown that the magnitude of normal tissue sparing by FLASH is typically modulated by irradiations on time scales of ≲1 s (see Figure 2a–c).^4^, ^24^, ^25^ The observed key dependencies and relevant time scales for the modulation of the FLASH normal tissue sparing magnitude suggest that the radiosensitivity of the biological system behaves as if it is transiently pushed away from its conventional state by swift dose delivery. Such behavior can be effectively modeled using the framework of perturbed equilibrium state dynamics (ESD). For an effect description based on ESD, we introduce a generic latent quantity Q(t), which represents a pool of chemical or biological ‘resources’ that modulates the instantaneous relative radiosensitivity $S_{\mathrm{rel}}\left(Q\right)$ of the biological system during irradiation relative to a low dose rate irradiation. $S_{\mathrm{rel}}\left(Q\right)$ is a dimensionless scaling factor representing the relative weight of incremental dose contributions under perturbed equilibrium versus steady state $Q_{0}$. Q(t) may be pushed away from its equilibrium state $Q_{0}$ by irradiation with an instantaneous dose rate $\dot{D}\left(t\right)$ inducing a change Δ(Q) and recovers toward equilibrium at rate $\dot{R}\left(Q\right)$, reflecting ‘replenishment’ processes. Such a behavior is illustrated in Figure 2d. Not considering any spatial components, the dynamics of such a system can be described by a 1st‐order ordinary differential equation (ODE)

(2) $$\frac{dQ}{dt}=\Delta \left(Q\left(t\right)\right)\dot{D}\left(t\right)+\dot{R}\left(Q\left(t\right)\right)$$

with the boundary condition $Q\left(t=0\right)=Q_{0}$ and a system state between equilibrium and complete saturation/depletion, i.e. $Q_{0}\geq Q\left(t\right)\geq 0$. While not at equilibrium, Q(t) may alter the instantaneous relative radiosensitivity $S_{\mathrm{rel}}\left(Q\right)$ of the biological system between 1 and a minimum $S_{\min}$, with $S_{\mathrm{rel}}\left(t=0\right)=1$ and $S_{\min}>0$. The dose D delivered during the irradiation session of time T is given by

(3) $$D=\int_{0}^{T}\dot{D}\left(t\right)dt$$

Assuming that radiobiological damage is altered by the status of Q(t) during dose delivery, the relative radiosensitivity (RRS)‐weighted dose $D_{\mathrm{RRS}}$ provides a measure for the effective radiobiological damage to the system compared to an irradiation at equilibrium

(4) $$D_{\mathrm{RRS}}=\int_{0}^{T}S_{\mathrm{rel}}\left(Q\left(t\right)\right)\;\dot{D}\left(t\right)dt$$

(5) $$\mathrm{RRS}=\frac{D_{\mathrm{RRS}}}{D}$$

In this study, we evaluated multiple ESD models with different dependencies for Δ(Q), $\dot{R}\left(Q\right)$, and $S_{\mathrm{rel}}\left(Q\right)$ based on their ability to describe experimental FMF_exp_ values. For this purpose, combinations of simple behaviors for Δ(Q), $\dot{R}\left(Q\right)$, and $S_{\mathrm{rel}}\left(Q\right)$ were evaluated as detailed in Table 2. Those behaviors are compatible with the previously discussed observations of FLASH normal tissue sparing and boundary conditions. For $S_{\mathrm{rel}}\left(Q\right)$, this included a step‐like behavior (RRS1), a power function (RRS2) that accommodates proportional as well as under‐ and over‐proportional behaviors, and a Michaelis–Menten‐type function (RRS3) that results in an intermediate behavior between RRS1 and a proportional behavior (i.e., under‐proportional), see Figure 2e. In the following, $Q_{0}$ was fixed to a generic value of 1. Because ODE ESD2 cannot reach Q=0, the combination ESD2_RRS1 was not evaluated, as it would leave RRS unchanged. Consequently, we evaluated in total eight combinations of ESD and RRS (see Tables 2 and 3) that had three or four free parameters. The tested functional forms were deliberately restricted to the simplest plausible dependencies (step‐like, linear, under/over‐proportional saturation/depletion responses), chosen to represent principal experimental trends while minimizing unnecessary complexity and reducing the risk of overfitting.

**TABLE 2:** Evaluated functional forms of (a) ordinary differential equations (ODE) (Equation 2) and (b) $S_{\mathrm{rel}}\left(Q\right)$.

(a)		
ODE ID	Δ(Q)	$\dot{R}\left(Q\right)$
ESD1	−gΘ(Q(t))	$r\;\Theta (Q_{0}-Q\left(t\right))$
ESD2	−gQ(t)	$r(Q_{0}-Q\left(t\right))$
ESD3	−gΘ(Q(t))	$r(Q_{0}-Q\left(t\right))$

(b)		
RRS ID	$S_{\mathrm{rel}}\left(Q\right)$	Comment
RRS1	$S_{\min}+\left(1-S_{\min}\right)\Theta \left(Q\left(t\right)/Q_{0}\right)$	Heaviside behavior
RRS2	$S_{\min}+\left(1-S_{\min}\right){\left(Q\left(t\right)/Q_{0}\right)}^{n}$	Power function behavior
RRS3	$S_{\min}+\left(1-S_{\min}\right)\;\frac{\left(1+K\right)Q\left(t\right)/Q_{0}\;}{K+Q\left(t\right)/Q_{0}}$	Michaelis–Menten type behavior

Abbreviations: Θ, Heaviside step function with Θ(x>0)=1 and Θ(x≤0)=0; const, constant; ESD, equilibrium state dynamics; RRS, relative radiosensitivity.

**TABLE 3:** Goodness of fit evaluations for all eight evaluated combinations of equilibrium state dynamics (ESD) and relative radiosensitivity (RRS) $S_{\mathrm{rel}}$.

Model ID^a)^	$R^{2}$	MAE	MAPE	RMSE	BIC	ΔBIC	Optimized parameters
ESD1_RRS1	0.911	0.034	4.3%	0.043	−314.7	0.0	3
ESD1_RRS2	0.910	0.033	4.2%	0.043	−310.7	4.0	4
ESD1_RRS3	0.909	0.034	4.3%	0.044	−310.1	4.6	4
ESD2_RRS2	0.917	0.032	4.1%	0.042	−314.4	0.3	4
ESD2_RRS3	0.911	0.034	4.3%	0.043	−311.3	3.4	4
ESD3_RRS1	0.908	0.034	4.3%	0.044	−313.3	1.4	3
ESD3_RRS2	0.907	0.034	4.3%	0.044	−308.9	5.8	4
ESD3_RRS3	0.908	0.034	4.3%	0.044	−309.1	5.6	4

*Note*: For details, see Equation 2, Table 2, and corresponding text. $Q_{0}$ was always fixed to 1.

Abbreviations: BIC, Bayesian Information Criterion; MAE, mean absolute error; MAPE, mean absolute percentage error; $R^{2},$ coefficient of determination; RMSE, root mean squared error.

^a)^ ESD2_RRS1 was discarded upfront because FMF_pred_ is always 1 regardless of delivery pattern (details see text).

Isoeffective dose ratios predicted by ESD models were calculated as

(6) $$\mathrm{FM}F_{\mathrm{pred}}={\left.\frac{\mathrm{RR}S_{\mathrm{test}}\;}{\;\mathrm{RR}S_{\mathrm{ref}}\;}\right|}_{\mathrm{isoeffect}}$$

as follows from Equation 1 and isoeffectiveness (i.e., $\mathrm{RR}S_{\mathrm{test}}\;D_{\mathrm{test}}=\mathrm{RR}S_{\mathrm{ref}}\;D_{\mathrm{ref}}$). RRS‐weighted dose $D_{\mathrm{RRS}}$, RRS and $\mathrm{FM}F_{\mathrm{pred}}$ (Equations (2), (3), (4), (5), (6)) were computed by numerically solving the corresponding ODE and integrals for the respective experimental temporal dose delivery profiles $\dot{D}\left(t\right)$. For this purpose, experimental irradiation profiles $\;\dot{D}\left(t\right)$ were calculated at the dosimetric reference points. Broad beam electron irradiations were spatiotemporally constant^18^ and temporal structure of UHDR dose delivery was derived from the temporal pulse beam structure parameters and the total dose while assuming rectangular pulses, see Table S1. For scanned proton irradiations, $\;\dot{D}\left(t\right)$ at the field center was calculated based on simulated intensities and delivery times of each spot, its distance to the field center and the spot scan order, while taking into account the non‐Gaussian spot dose profile previously measured in vivo during the experiments.^27^ $\dot{D}\left(t\right)$ of reference dose rate irradiations were approximated by a continuous irradiation at constant dose rates of 0.11 and 0.37 Gy/s for datasets e1 and p1 (Table 1), respectively.

### 2.5 Model fitting, evaluation, and ranking

The eight ESD model variants (see Tables 2 and 3) were evaluated based on their capability to fit the collection of experimental $\mathrm{FM}F_{\exp}$. The mean squared error objective function

(7) $$\chi^{2}=\sum_{i=1}^{n}{\left(\mathrm{FM}F_{\exp,\;i}-\mathrm{FM}F_{\mathrm{pred},\;i}\right)}^{2}/n$$

was optimized with the Nelder–Mead simplex algorithm^28^ (scipy.optimize.minimize v1.6.2). Model ranking was performed using the Bayesian Information Criterion (BIC) with ΔBIC being the BIC difference between a given model and the best‐performing model.^29^, ^30^ ΔBIC values of [0–2], [2–6], and [6–10] are referred to hereafter as *weak*, *positive*, and *strong* support for model rejection, respectively.^30^ The coefficient of determination ($R^{2}$), the mean absolute error (MAE), the mean absolute percentage error (MAPE), and the root mean squared error (RMSE) were evaluated as additional goodness‐of‐fit indicators. The Shapiro–Wilk test was used to assess normal distribution of the residuals with a significance level of 0.05.

## 3 RESULTS

**FIGURE 3:**
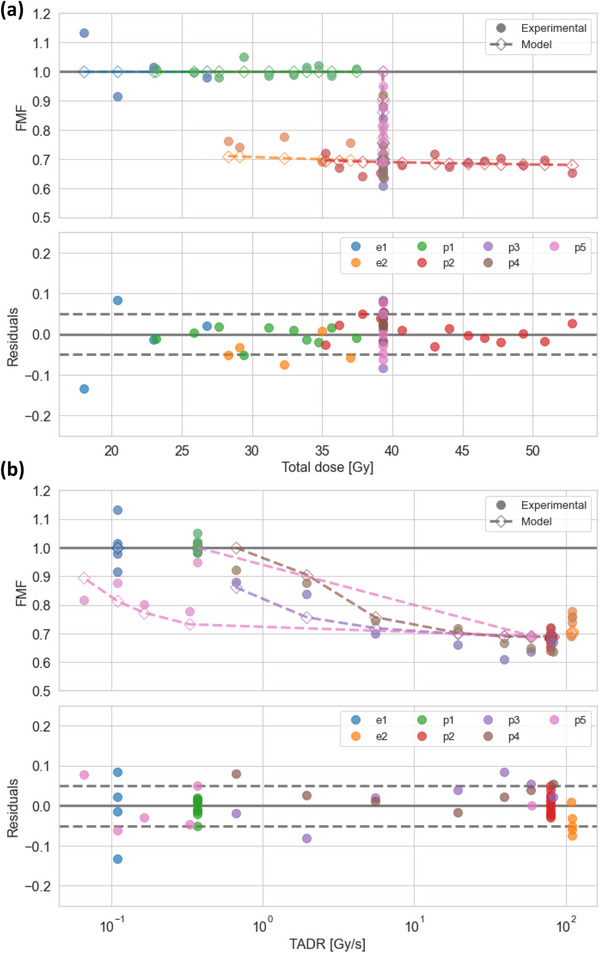
Simulated and experimental isoeffective dose ratios (FLASH‐modifying factors, FMF) and corresponding residuals for model variant ESD1_RRS1 as a function of dose (a) and as a function of time‐averaged dose rate (TADR) (b). For the residuals, dashed horizontal lines indicate ± 0.05.

**FIGURE 4:**
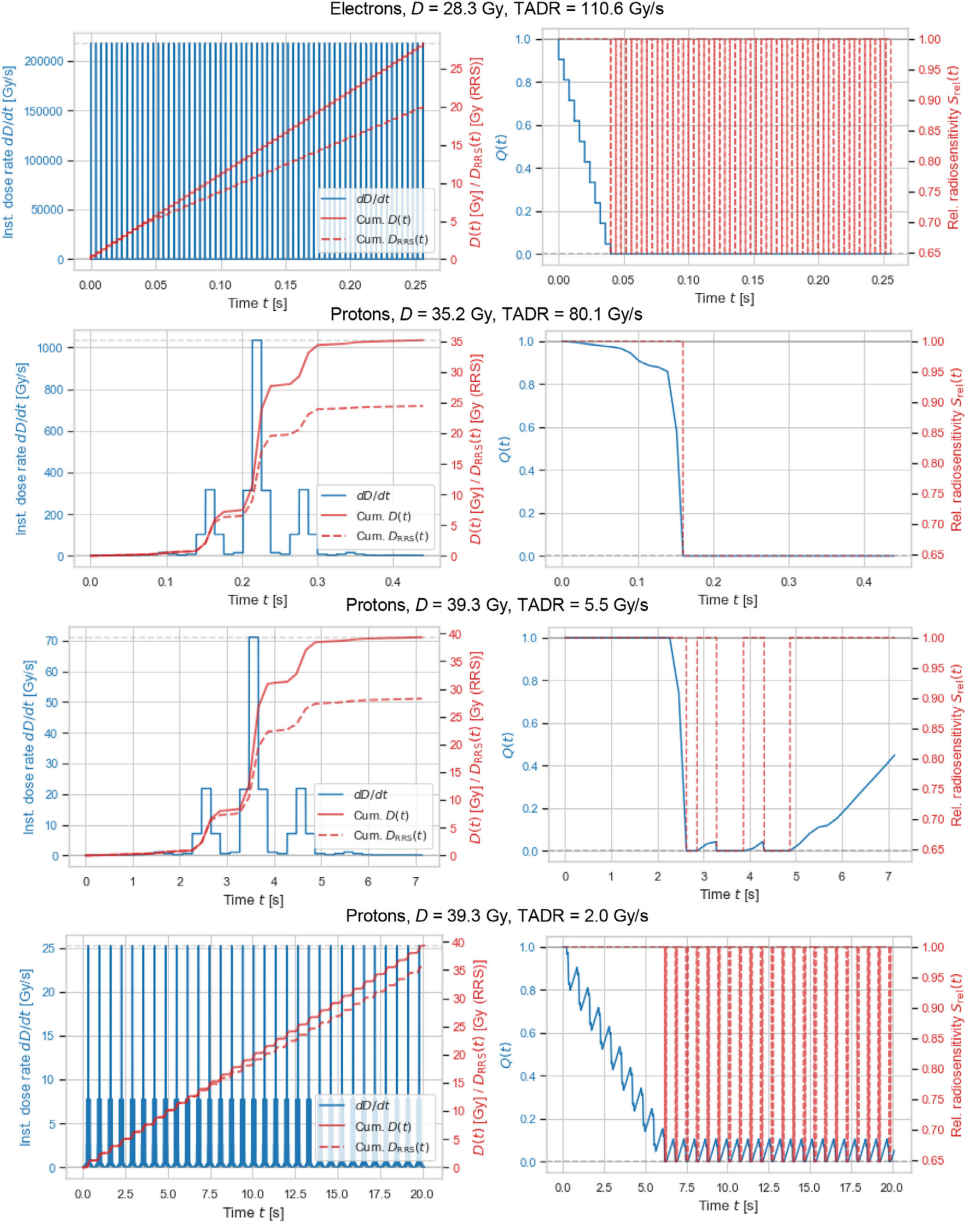
Four representative temporal irradiation profiles from the data collection are shown (left) alongside the simulated dynamics for 𝑄(𝑡) and 𝑆_rel_(𝑡) for the ESD1_RRS1 model variant (right). The corresponding accumulated RRS‐weighted dose, 𝐷_RRS_(𝑡), is also displayed (left).

Dose‐response curves for electron irradiations (e1 and e2) are shown in Figure 1b. A total of 123 experimental FMF_exp_ values were derived from the experimental data collection (Table 1) and resulted in 52 FMF_exp_ values after averaging FMF_exp_ values obtained from the same mouse group for different toxicity levels (Figure 3). Model variant ESD1_RRS1 was ranked best based on BIC, see Table 3, with $g=0.22\;Gy^{-1},\;r=0.29\;s^{-1},$ and $S_{\min}=0.65$. ΔBIC indicated *weak* support for model rejection for ESD2_RRS2 and ESD3_RRS1, whereas *positive* support was indicated for the remaining five model variants. All eight candidate ESD models were able to reproduce experimental FMF_exp_ values with good accuracy ($R^{2}$ within 0.907–0.917, MAE within 0.032–0.034, MAPE within 4.1–4.3%, and RMSE within 0.042–0.044) using three or four free parameters, see Table 3, and resulted in similar values for $S_{\min}$ (range: 0.55–0.65). Simulated FMF_pred_ and experimental FMF_exp_ values and corresponding residuals for the best ranked model are shown in Figure 3a,b as a function of dose and TADR, respectively. The Shapiro–Wilk test statistic for residuals was 0.98 with *p* = 0.4, indicating no significant deviation from normality. Variation of residuals were similar for the reference data sub‐group (e1, p1: MAE=0.030, RMSE=0.046) and the non‐reference data sub‐group (e2, p2, p3, p4, p5: MAE=0.035, RMSE=0.042), suggesting that residuals are driven by uncertainties in the experimental data, rather than by inabilities of the model to capture key data trends. Figure 4 displays examples of temporal irradiation profiles together with the simulated dynamics for Q(t), $S_{\mathrm{rel}}\left(t\right)$, and the corresponding accumulated RRS‐weighted dose $D_{\mathrm{RRS}}\left(t\right)$ for the selected model variant ESD1_RRS1.

## 4 DISCUSSION

Equilibrium state dynamics‐based modeling offers a parsimonious, data‐driven, and mechanistically agnostic framework that generalizes established dose‐modifying concepts (e.g., OER‐weighted dose) to describe FLASH normal tissue sparing quantitatively, emphasizing the role of transient equilibrium perturbations in modulating radiosensitivity. Unlike our previous work focusing only on single‐fraction dose dependence,^1^ the present study extends to capture key experimental dependencies on temporal structure using only three or four free parameters in a simple first‐order ODE. The limited parameterization inherent to ESD combined with its simplicity addresses common challenges in radiobiological modeling, such as overfitting of sparse data, and provides a framework for parameter extraction from experimental studies. ESD does not assume a priori that the temporal dose delivery structure on a certain time scale determines FLASH sparing. Instead, the modeling framework allows such dependencies to emerge naturally from the fitted parameters if supported by data. ESD models were shown in this work to be able to describe consistently an extensive collection of experimental isoeffect dose ratios (FMF_exp_), spanning diverse temporal dose delivery patterns, as well as pulsed electron and scanned proton beams, covering thereby a large domain of relevance for the clinical transfer of FLASH‐RT. The best‐performing model ESD1_RRS1 could reproduce experimental FMF with an MAE of 0.034 using three free parameters.

If the extent of FLASH normal tissue sparing is modulated according to ESD, this holds important implications for its clinical transfer. It underscores the critical importance of delivering hypo‐ or even extremely hypofractionated FLASH‐RT in order to maximize normal tissue sparing by FLASH, assuming equitoxicity for tumor tissues.^31^ However, in many clinical scenarios, such fractionation schemes may compromise the sparing of late‐reacting tissues.^32^, ^33^, ^34^ Furthermore, it suggests that any FLASH‐RT fraction should be delivered on time scales well below recovery times of critical normal tissues to maximize their sparing. A linear recovery of $r=0.29\;s^{-1}$ was obtained for model variant ESD1_RRS1 for acute murine skin reactions, though recovery times likely vary by tissue and endpoint.

Out of the eight evaluated ESD‐based models, all reproduced the experimental isoeffect dose ratios (FMF_exp_) with good accuracy using three or four free parameters (Table 3). Models with four parameters achieved similar RMSE and MAE compared to three‐parameter variants but generally had higher BIC values, indicating mild overfitting—apart from ESD2_RRS2, which performed comparably. The fact that several variants yielded similarly low BIC values highlights that the dataset lacks sufficient discriminative power to decisively favor one functional form, reflecting both the limited precision and domain of the data, as well as similarities between the tested functional dependencies (e.g., Michaelis–Menten vs. factor‐type forms). Within this context, ESD1_RRS1 can be regarded as a straightforward and practical three‐parameter model for capturing the primary temporal dose‐delivery dependencies of acute murine FLASH skin sparing. However, a constant change Δ(*Q*) and recovery $\dot{R}\left(Q\right)$ combined with a non‐continuous, step‐like $S_{\mathrm{rel}}\left(Q\right)$ may be overly simplistic, and future investigations should refine these behaviors to provide a more accurate representation and investigate predictive performances for unseen data.

ESD1_RRS1 (Equations (3), (4), (5), (6) and Table 2) reduces for irradiations at a constant dose rate ${\dot{D}}_{c}$ with $g{\dot{D}}_{c}>r$ to

(8) $$\begin{array}{cc} & \mathrm{RRS}=D_{\mathrm{RRS}}/D\\ & =\left\{\begin{array}{cc} 1 & \mathrm{for}\;T<\;Q_{0}/\left(g{\dot{D}}_{c}-r\right)\\ \left(1-S_{\min}\right)\frac{{\dot{D}}_{c}Q_{0}}{D\left(g{\dot{D}}_{c}-r\right)}+S_{\min} & \mathrm{for}\;T\;\geq Q_{0}/\left(g{\dot{D}}_{c}-r\right) \end{array}\right. \end{array}$$

and, similarly, for irradiations for which exposure times T are short so that recovery r is negligible, it follows

(9) $$\mathrm{RRS}=D_{\mathrm{RRS}}/D=\left\{\begin{array}{cc} 1 & \mathrm{for}\;D<\;Q_{0}/g\\ \left(1-S_{\min}\right)\frac{Q_{0}}{Dg}+S_{\min} & \mathrm{for}\;D\;\geq Q_{0}/g \end{array}\right.$$

Pulsed irradiations with many pulses can be approximated by the constant dose rate case and representations similar to Equations 8 and 9 can also be obtained for other ESD model variants for high doses and in some cases also for lower doses. Assuming a $\mathrm{RR}S_{\mathrm{ref}}$ (Equations (4), (5), (6)) of 1, Equations 8 and 9 are both equivalent to the SET function, which was shown to describe the single fraction dose‐dependency of FLASH normal tissue sparing for multiple experimental datasets in a recent meta‐analysis.^1^ For $\mathrm{RR}S_{\mathrm{ref}}=1$, the following association with parameters of the SET function can be made: $\mathrm{FM}F^{\min}=S_{\min}$ and $\;D_{T}=\frac{{\dot{D}}_{c}Q_{0}}{\left(g{\dot{D}}_{c}-r\right)}$. For a negligible r, this reduces to $\;D_{T}=\frac{Q_{0}}{g}$. A $\mathrm{FM}F^{\min}$ of 0.67 and 0.51 was found for all pooled data and for skin data, respectively.^1^ This is in reasonable agreement with $S_{\min}=0.65$, as determined in this study for model ESD1_RRS1. In contrast, the $\;D_{T}$ values of 9.6 Gy (all pooled data) and 16.6 Gy (skin data) found by the meta‐analysis are considerably higher than a $\;D_{T}$ of 4.3 Gy obtained by ESD1_RRS1 for a negligible r and $\mathrm{RR}S_{\mathrm{ref}}=1$ (Equation 9). This might be attributed to limitations when extrapolating the ESD model to lower dose regions for which it was not fitted.

In this study, ESD was fitted and evaluated exclusively on acute murine skin reactions obtained with pulsed electron beams and scanned proton beams. Although the framework is general and broadly adaptable, its parameters are context‐specific and not assumed to transfer across tissues, endpoints, or species. Re‐fitting will be required for new applications. Consequently, claims of generalizability are limited, and any potential clinical translation remains speculative pending validation with independent datasets. ESD models are bound to the data domain for which they have been validated, and as a phenomenological modeling approach, they do not rule out that additional, complementary processes may further influence normal tissue sparing by FLASH. For instance, in contrast to the electron and proton skin toxicity data utilized in this work, recent studies identified considerable differences in intestinal responses to UHDR proton and electron irradiation^35^ and suggest a strong biological component in the mechanisms of the FLASH sparing effect for different systems and endpoints.^21^ The absence of a sparing effect for tumoral tissues is also not directly deducible from ESD models. However, while these aspects and differences cannot be explained by the ESD‐based modeling approach presented here, they can in principle be incorporated by endpoint‐ and modality‐specific model parameters. Furthermore, we have omitted explicit spatial recovery dynamics (e.g., diffusion) due to a lack of suitable data, even though such spatial factors are likely to affect FLASH sparing when irradiation and system recovery times occur on comparable time scales (∼1s).

ESD‐based models obtained in this study are compatible with hypotheses that sub‐second physical and chemical processes may contribute to the mechanism of the FLASH effect, such as radical recombination, reactive oxygen species scavenging, lipid peroxidation, and Fenton chemistry, as well as oxygen depletion and recovery.^7^, ^8^, ^9^, ^36^ Taken together, the ESD‐based data‐driven description of FLASH sparing aligns with a saturation or depletion phenomenon that modifies the instantaneous radiosensitivity and that has for acute murine skin reactions a characteristic recovery rate on a sub‐second time scale. In fact, as a phenomenological modeling approach, ESD may help narrow down the search for eligible mechanisms of action for the FLASH effect and may serve as a forerunner and benchmark for more mechanistic/process‐oriented descriptions. Although ESD models reproduced the acute murine skin dataset with high fidelity, the inability to decisively favor one variant over another limits the extent to which biological inferences can be drawn. This underscores that current datasets are better suited for phenomenological description than for mechanistic discrimination. Future experiments should therefore be designed to probe parameter‐sensitive regimes, for example via split‐dose studies with controlled gap times, different temporal beam structure parameters, such as a varying pulse repetition frequency, or intermediate dose ranges where predictions diverge. Incorporating high‐precision readouts and cross‐tissue validations may further increase discriminative power.

It should be underscored that ESD‐based modeling is mathematically similar to a description of FLASH normal tissue sparing based on the conventional radiolytic oxygen depletion hypothesis^10^, ^11^ that is difficult to reconcile with oxygen depletion measurements and modeling,^12^, ^13^, ^14^ as initially mentioned. In fact, the model variant ESD2_RRS3 in the current study is mathematically identical to the OER‐weighted dose model, which has already previously been shown to describe the presented proton dataset successfully,^10^ however, with a different naming of the involved parameters (*O*(*t*), *g*, *λ*, *O*_env_, OER/OER_env_, 1/OER_env_, *K*/*O*_env_, and *m* in Ref. 10 correspond to *Q*(*t*), *g*, *r*, *Q*_0_, *S*_rel_, *S*_min_, *K*, and 1/*S*_min_(1+*K*)‐*K* in ESD2_RRS3). Despite yielding similar residuals, ESD2_RRS3 was penalized by the BIC because of its larger number of free parameters and was therefore less favored than ESD1_RRS1. The novelty of the current study lies in extending the experimental dataset with pulsed electron beam measurements and demonstrating that a broader variety of ESD‐based models can reproduce the experimental data consistently. Furthermore, the ESD‐based description and corresponding model parameters as formulated in this work are not attached to specific mechanistic processes, such as oxygen depletion.

The functional forms used to link the latent quantity *Q* to radiosensitivity were deliberately selected to represent the simplest plausible dependencies that are consistent with experimental observations: a step function to mimic a threshold‐like response and power‐law and Michaelis–Menten‐type forms to capture proportional or supra‐/sub‐proportional effects and to represent saturation/depletion behavior. These forms are widely used across biological modeling because they balance descriptive power with parsimony, and they allow the data to identify whether responses behave more like thresholds, proportional shifts, or saturating processes. By starting with these minimal formulations, the ESD framework avoids introducing unnecessary mechanistic assumptions while still providing flexibility to capture the principal experimental trends. In this sense, the purpose of the model is not to prescribe a single mechanistic explanation but to offer a parsimonious, phenomenological abstraction that can unify different temporal‐dose dependencies within one consistent mathematical framework. Such abstractions may support hypothesis generation, enable quantitative comparison across datasets, and help design experiments that can further test where different functional forms diverge.

## 5 CONCLUSIONS

ESD‐based modeling can capture key temporal dose‐delivery dependencies of FLASH acute skin sparing using a parsimonious, data‐driven approach. Further investigations using independent datasets are needed to assess the predictive performances of ESD and whether modeled behaviors extend to other tissues, endpoints, modalities, and delivery patterns. If these behaviors generalize, ESD‐based modeling could serve as a practical framework for evaluating and optimizing UHDR irradiations in preclinical and translational settings.

## CONFLICT OF INTEREST STATEMENT

The authors declare no conflict of interest.

## HUMAN/ANIMAL REGULATORY APPROVAL

For new murine electron data, published first within this study: the Animal Ethics Committee of Vaud, Switzerland (Approval No. VD3934 and VD3962). For previously published research data that are used by this study, the respective publications apply.

## Supporting information



Supporting Information

## Data Availability

New research data are stored in an institutional repository and will be shared upon reasonable request to the corresponding author. For previously published research data, the respective publications apply.

## References

[1] Böhlen TT , Germond JF , Bourhis J , et al. Normal tissue sparing by FLASH as a function of single‐fraction dose: a quantitative analysis. Int J Radiat Oncol. 2022;114(5):1032‐1044. Available from: https://linkinghub.elsevier.com/retrieve/pii/S0360301622005417 10.1016/j.ijrobp.2022.05.038 35810988

[2] Sørensen BS , Kanouta E , Ankjærgaard C , et al. Proton FLASH: impact of dose rate and split dose on acute skin toxicity in a murine model. Int J Radiat Oncol. 2024;120:265‐275. Available from: https://linkinghub.elsevier.com/retrieve/pii/S0360301624006217 10.1016/j.ijrobp.2024.04.071 38750904

[3] Mascia A , McCauley S , Speth J , et al. Impact of multiple beams on the FLASH effect in soft tissue and skin in mice. Int J Radiat Oncol. 2024;118(1):253‐261. Available from: https://linkinghub.elsevier.com/retrieve/pii/S0360301623076782 10.1016/j.ijrobp.2023.07.024 37541394

[4] Ruan JL , Lee C , Wouters S , et al. Irradiation at ultra‐high (FLASH) dose rates reduces acute normal tissue toxicity in the mouse gastrointestinal system. Int J Radiat Oncol. 2022;111(5):1250‐1261. Available from: https://linkinghub.elsevier.com/retrieve/pii/S0360301621026432 10.1016/j.ijrobp.2021.08.004 PMC7612009 34400268

[5] Vozenin MC , Bourhis J , Durante M . Toward clinical translation of FLASH radiotherapy. Nat Rev Clin Oncol. 2023;19(12):791‐803. Available from: https://www.nature.com/articles/s41571‐022‐00697‐z 10.1038/s41571-022-00697-z 36303024

[6] Grilj V , Zayas AV , Sesink A , et al. Average dose rate is the major temporal beam structure parameter for preserving murine intestines with pulsed electron FLASH‐RT. Int J Radiat Oncol. 2025;123:593‐601. Available from: https://linkinghub.elsevier.com/retrieve/pii/S036030162500389X 10.1016/j.ijrobp.2025.04.021 40319927

[7] Chow JCL , Ruda HE . Mechanisms of action in FLASH radiotherapy: a comprehensive review of physicochemical and biological processes on cancerous and normal cells. Cells. 2024;13(10):835. Available from: https://www.mdpi.com/2073‐4409/13/10/835 38786057 10.3390/cells13100835 PMC11120005

[8] Friedl AA , Prise KM , Butterworth KT , Montay‐Gruel P , Favaudon V . Radiobiology of the FLASH effect. Med Phys. 2021;49(3):1993‐2013. Available from: https://onlinelibrary.wiley.com/doi/10.1002/mp.15184 34426981 10.1002/mp.15184

[9] Limoli CL , Vozenin MC . Reinventing radiobiology in the light of FLASH radiotherapy. Annu Rev Cancer Biol. 2023;7(1):1‐21. Available from: https://www.annualreviews.org/doi/10.1146/annurev‐cancerbio‐061421‐022217 39421564 10.1146/annurev-cancerbio-061421-022217 PMC11486513

[10] Poulsen PR , Johansen JG , Sitarz MK , et al. Oxygen enhancement ratio weighted dose quantitatively describes acute skin toxicity variations in mice after pencil beam scanning proton FLASH irradiation with changing doses and time structures. Int J Radiat Oncol. 2024;120(1):276‐286. Available from: https://linkinghub.elsevier.com/retrieve/pii/S0360301624003584 10.1016/j.ijrobp.2024.02.050 38462015

[11] Petersson K , Adrian G , Butterworth K , McMahon SJ . A quantitative analysis of the role of oxygen tension in FLASH radiation therapy. Int J Radiat Oncol. 2023;107(3):539‐547. Available from: https://linkinghub.elsevier.com/retrieve/pii/S0360301620308841 10.1016/j.ijrobp.2020.02.634 32145319

[12] Boscolo D , Scifoni E , Durante M , Krämer M , Fuss MC . May oxygen depletion explain the FLASH effect? A chemical track structure analysis. Radiother Oncol. 2021;162:68‐75. Available from: https://linkinghub.elsevier.com/retrieve/pii/S0167814021066184 34214612 10.1016/j.radonc.2021.06.031

[13] Cao X , Zhang R , Esipova TV , et al. Quantification of oxygen depletion during FLASH irradiation in vitro and in vivo. Int J Radiat Oncol Biol Phys. 2021;111(1):240‐248. doi:10.1016/j.ijrobp.2021.03.056 33845146 10.1016/j.ijrobp.2021.03.056 PMC8338745

[14] Jansen J , Knoll J , Beyreuther E , et al. Does FLASH deplete oxygen? Experimental evaluation for photons, protons, and carbon ions. Med Phys. 2021;48(7):3982‐3990. Available from: https://onlinelibrary.wiley.com/doi/10.1002/mp.14917 33948958 10.1002/mp.14917

[15] Ingalls B . Mathematical Modelling in Systems Biology: An Introduction. MIT Press; 2014.

[16] Smith HL , Waltman PE . The Theory of the Chemostat: Dynamics of Microbial Competition. Cambridge University Press; 1995. doi:10.1017/CBO9780511530043

[17] Jaccard M , Durán MT , Petersson K , et al. High dose‐per‐pulse electron beam dosimetry: commissioning of the Oriatron eRT6 prototype linear accelerator for preclinical use: commissioning. Med Phys. 2018;45(2):863‐874. doi:10.1002/mp.12713 29206287 10.1002/mp.12713

[18] Böhlen TT , Psoroulas S , Aylward JD , et al. Recording and reporting of ultra‐high dose rate “FLASH” delivery for preclinical and clinical settings. Radiother Oncol. 2024;200:110507. Available from: https://linkinghub.elsevier.com/retrieve/pii/S0167814024007771 39245070 10.1016/j.radonc.2024.110507

[19] Sørensen BS , Krzysztof Sitarz M , Ankjærgaard C , et al. In vivo validation and tissue sparing factor for acute damage of pencil beam scanning proton FLASH. Radiother Oncol. 2022;167:109‐115. doi:10.1016/j.radonc.2021.12.022 34953933 10.1016/j.radonc.2021.12.022

[20] ICRU. ICRU Report 93: prescribing, recording, and reporting light ion beam therapy. J ICRU 2019;16(1–2):5‐36.

[21] Horst F , Bodenstein E , Brand M , et al. Dose and dose rate dependence of the tissue sparing effect at ultra‐high dose rate studied for proton and electron beams using the zebrafish embryo model. Radiother Oncol. 2024;194:110197. Available from: https://linkinghub.elsevier.com/retrieve/pii/S0167814024001191 38447870 10.1016/j.radonc.2024.110197

[22] Horst F , Brand M , Hans S , et al. Zebrafish embryo model of the FLASH effect. Regard to Böhlen. J Int J Radiat Oncol Biol Phys. 2023;115(4):1006‐1007. doi:10.1016/j.ijrobp.2022.11.015 36822772

[23] Böhlen TT , Germond JF , Bochud F , et al. In reply to Horst et al. Int J Radiat Oncol. 2023;115(4):1007‐1009. Available from: https://www.sciencedirect.com/science/article/pii/S0360301622035301 10.1016/j.ijrobp.2022.11.018 36822773

[24] Montay‐Gruel P , Petersson K , Jaccard M , et al. Irradiation in a flash: unique sparing of memory in mice after whole brain irradiation with dose rates above 100 Gy/s. Radiother Oncol. 2017;124(3):365‐369. doi:10.1016/j.radonc.2017.05.003 28545957 10.1016/j.radonc.2017.05.003

[25] Kacem H , Almeida A , Cherbuin N , Vozenin MC . Understanding the FLASH effect to unravel the potential of ultra‐high dose rate irradiation. Int J Radiat Biol. 2021;98(3):506‐516. doi:10.1080/09553002.2021.2004328 34788193

[26] Montay‐Gruel P , Petersson K , Jaccard M , et al. Irradiation in a flash: unique sparing of memory in mice after whole brain irradiation with dose rates above 100 Gy/s. Radiother Oncol. 2017;124(3):365‐369. Available from: 10.1016/j.radonc.2017.05.003 28545957

[27] Kanouta E , Poulsen PR , Kertzscher G , Sitarz MK , Sørensen BS , Johansen JG . Time‐resolved dose rate measurements in pencil beam scanning proton FLASH therapy with a fiber‐coupled scintillator detector system. Med Phys. 2024;50(4):2450‐2462. Available from: https://aapm.onlinelibrary.wiley.com/doi/10.1002/mp.16156 10.1002/mp.16156 36508162

[28] Gao F , Han L . Implementing the Nelder‐Mead simplex algorithm with adaptive parameters. Comput Optim Appl. 2024;51(1):259‐277. http://link.springer.com/10.1007/s10589‐010‐9329‐3

[29] Burnham KP , Anderson DR . Multimodel inference: understanding AIC and BIC in model selection. Sociol Methods Res. 2025;33(2):261‐304. Available from: https://journals.sagepub.com/doi/10.1177/0049124104268644

[30] Raftery AE . Bayesian model selection in social research. Sociol Methodol. 2025;25:111. Available from: https://www.jstor.org/stable/271063?origin=

[31] Böhlen TT , Germond JF , Petersson K , et al. Effect of conventional and ultrahigh dose rate FLASH irradiations on preclinical tumor models: a systematic analysis. Int J Radiat Oncol. 2023;117(4):1007‐1017. Available from: https://linkinghub.elsevier.com/retrieve/pii/S0360301623005357 10.1016/j.ijrobp.2023.05.045 37276928

[32] Böhlen TT , Germond J , Bourhis J , Bailat C , Bochud F , Moeckli R . The minimal FLASH sparing effect needed to compensate the increase of radiobiological damage due to hypofractionation for late‐reacting tissues. Med Phys. 2023;49(12):7672‐7682. Available from: https://onlinelibrary.wiley.com/doi/10.1002/mp.15911 10.1002/mp.15911 PMC10087769 35933554

[33] Böhlen TT , Germond J , Bourhis J , et al. Technical note: break‐even dose level for hypofractionated treatment schedules. Med Phys. 2022;48(11):7534‐7540. Available from: https://onlinelibrary.wiley.com/doi/10.1002/mp.15267 10.1002/mp.15267 PMC9298418 34609744

[34] Unkelbach J , Craft D , Salari E , Ramakrishnan J , Bortfeld T . The dependence of optimal fractionation schemes on the spatial dose distribution. Phys Med Biol. 2023;58(1):159‐167. Available from: https://iopscience.iop.org/article/10.1088/0031‐9155/58/1/159 10.1088/0031-9155/58/1/159 23221166

[35] Liu K , Titt U , Esplen N , et al. Discordance in acute gastrointestinal toxicity between synchrotron‐based proton and linac‐based electron ultra‐high dose rate irradiation. Int. J. Radiat. Oncol. Biol. Phys. and Int J Radiat Oncol Biol Phys 2025;122(2):491‐501. 39862897 10.1016/j.ijrobp.2025.01.007 PMC13383351

[36] Weber UA , Scifoni E , Durante M . FLASH radiotherapy with carbon ion beams. Med Phys. 2022;49(3):1974‐1992. Available from: https://onlinelibrary.wiley.com/doi/10.1002/mp.15135 34318508 10.1002/mp.15135

