# Supplementary material for: Equilibrium state dynamics‐based modeling of temporal dose delivery dependencies of FLASH skin sparing
Source: Med Phys. 2025 Nov 21;52(12):e70143. doi: 10.1002/mp.70143 (PMC12638282; doi:10.1002/mp.70143)
Supplement: Supplementary file 1 — Supporting Information [file MP-52-0-s001.doc]

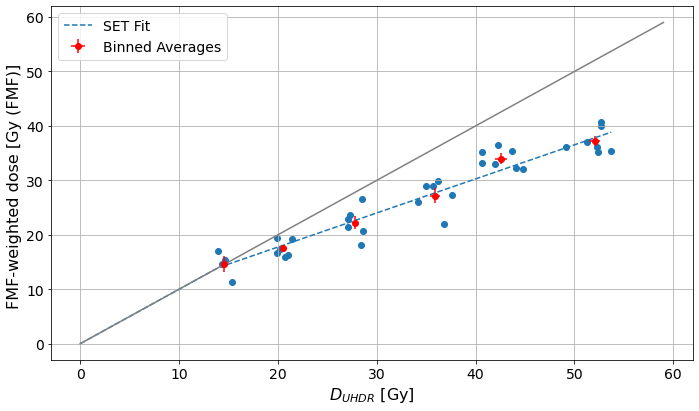

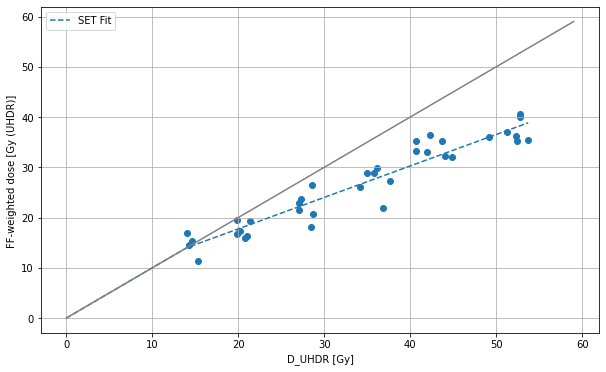


**Supplementary Figure 1:** Regression of the sudden effect transition (SET) function to data from Horst et al. ^[1,2]^. Abbreviations: FMF = FLASH-modifying factor, UHDR = Ultra-high dose rate.

**Supplementary Table 1:** Summary of key set-up and irradiation parameters for electron skin irradiations. Reporting follows recommendations for spatiotemporally constant irradiations using a beam centric approach ^[3]^.

| **Quantity** | **Abbreviation** | **Example values** |
| --- | --- | --- |
| **Minimal reporting** | | |
| *General description* | | |
| Device name | Oriatron eRT6 (PMB) | |
| Accelerator type | Electron linac | |
| Dose delivery technique | Scattering and collimation | |
| Traceability and dosimetry code of practice used | Gafchromic EBT-XD films and Advanced Markus ionization chamber (PTW) were traceably cross-calibrated in a clinical linac (Synergy, Elekta) at various conventional dose rate (CDR) electron energies in solid water slabs at clinical reference conditions (field size 10x10 cm^2^, SSD = 100 cm, detectors at D_max_ for the specific energy), following the TRS-398 protocol. | |
| Additional key information about delivery | Custom made 3 cm thick carbon collimator (10×10 cm^2^) with a 15×15 mm^2^ square aperture.  Both conventional and UHDR beam delivery based on total number of pulses and monitored by an ACCT detector. | |
| Preclinical: Biological system(s), model(s), endpoint(s) | Dorsal skin of BALB/c mice  NTCP scored as percentage of animals that develop large ulcers (> 3mm) over the monitoring time (50 d).  Anesthesia: ketamine | |
| Additional key information (including imaging) about irradiated systems/models/patients | - | |
| *Non-temporal beam parameters* | | |
| Radiation type and nominal beam energy | *E* | Electrons, 6 MeV for CDR, 5 MeV for UHDR |
| Beam dose at reference point | *D*_beam_ | 18, 20.4, 23, 26.8, 29.2, 31.9 Gy (CDR)  23.1, 26.1, 28.3, 29.1, 32.3, 35, 37 Gy (UHDR) |
| Reference point specification | *P*_ref_ | Thin film dose, film mounted in contact with the collimator. |
| Source-to-surface distance | SSD | 60 cm (CDR), 130 cm (UHDR) |
| Field size | FS | Square collimator measuring 15 mm x 15 mm. Total irradiated skin area measured 20 mm x 15 mm. |
| *Temporal beam structure parameters* | | |
| Pause before next beam | *ΔT*_beam_ | N/A |
| Beam-on time | *T*_beam_ | 165-290 s (CDR), 0.2-0.3 s (UHDR) |
| Number of pulses for beam | *#*pulse | ~1500-3000 (CDR), 59-89 (UHDR) |
| Pulse length | *t*_pulse_ | 1 µs (CDR), 2 µs (UHDR) |
| Pulse repetition frequency | *PRF* | 10 Hz (CDR), 250 Hz (UHDR) |
| Pulse charge | *Q*_pulse_ | N/A |
| Number of bunches per pulse | *#*bunch | 3E3 (CDR), 6E3 (UHDR) |
| Bunch length | *t*_bunch_ | N/A |
| Bunch repetition frequency | *BRF* | 3 GHz (S-band) |
| Bunch charge | *Q*_bunch_ | N/A |
| **Optimal reporting** | | |
| *Derived and additional parameters* | | |
| Average dose rate at reference point | ADR | 0.11 Gy s^-1^ (CDR), 110 Gy s^-1^ (UHDR) |
| Instantaneous dose rate at reference point | IDR | 0.011 MGy s^-1^ (CDR), 0.22 MGy s^-1^ (UHDR) |
| Dose per pulse | DPP | 0.011 Gy (CDR), 0.4 Gy (UHDR) |
| Representative 2D dose distribution of beam, PDD and lateral profiles | - | Percentage depth dose (SSD=30cm, EBT3 in solid water)  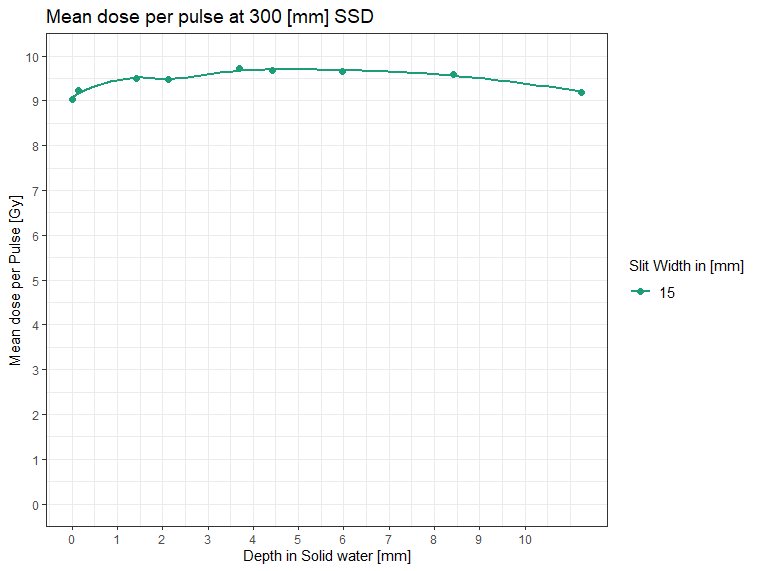  Horizontal dose profile for 15 mm x 15 mm field (SSD=30cm, EBT3 on solid water)  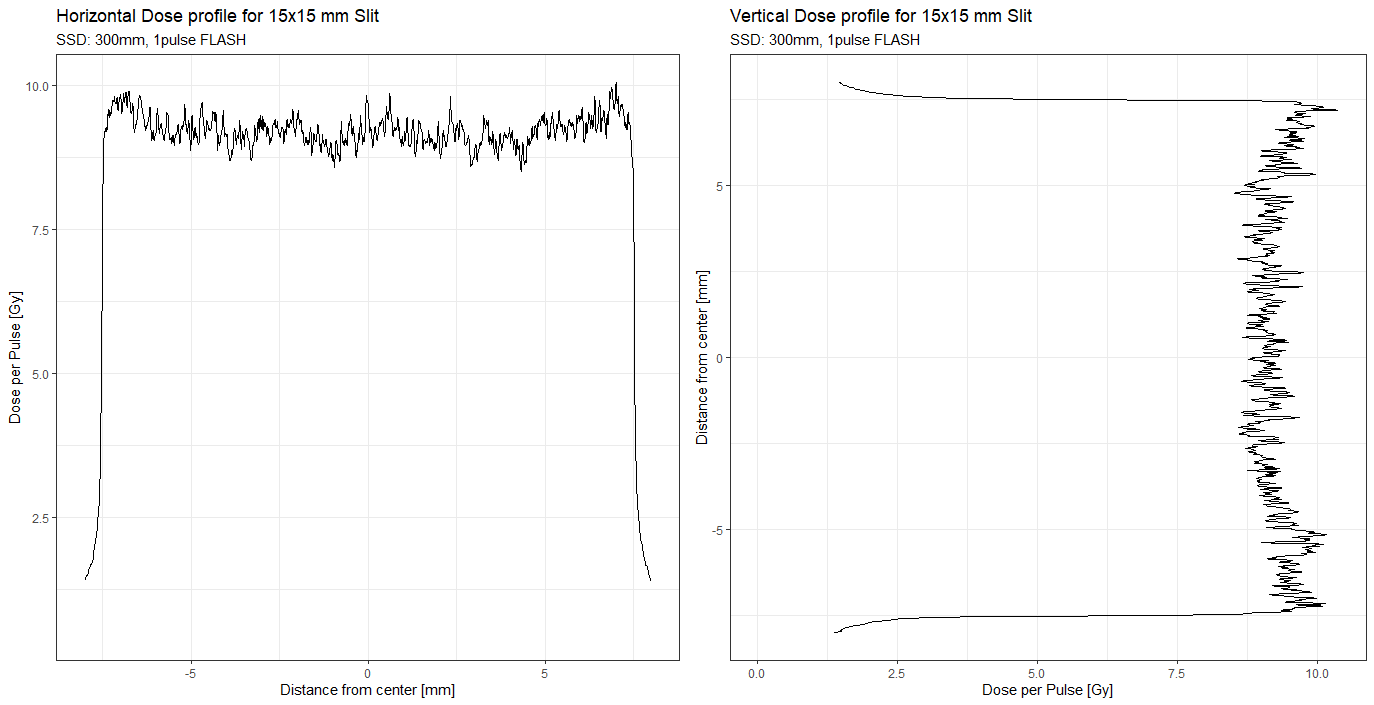  Horizontal dose profile (SSD=30cm, EBT3 on solid water)  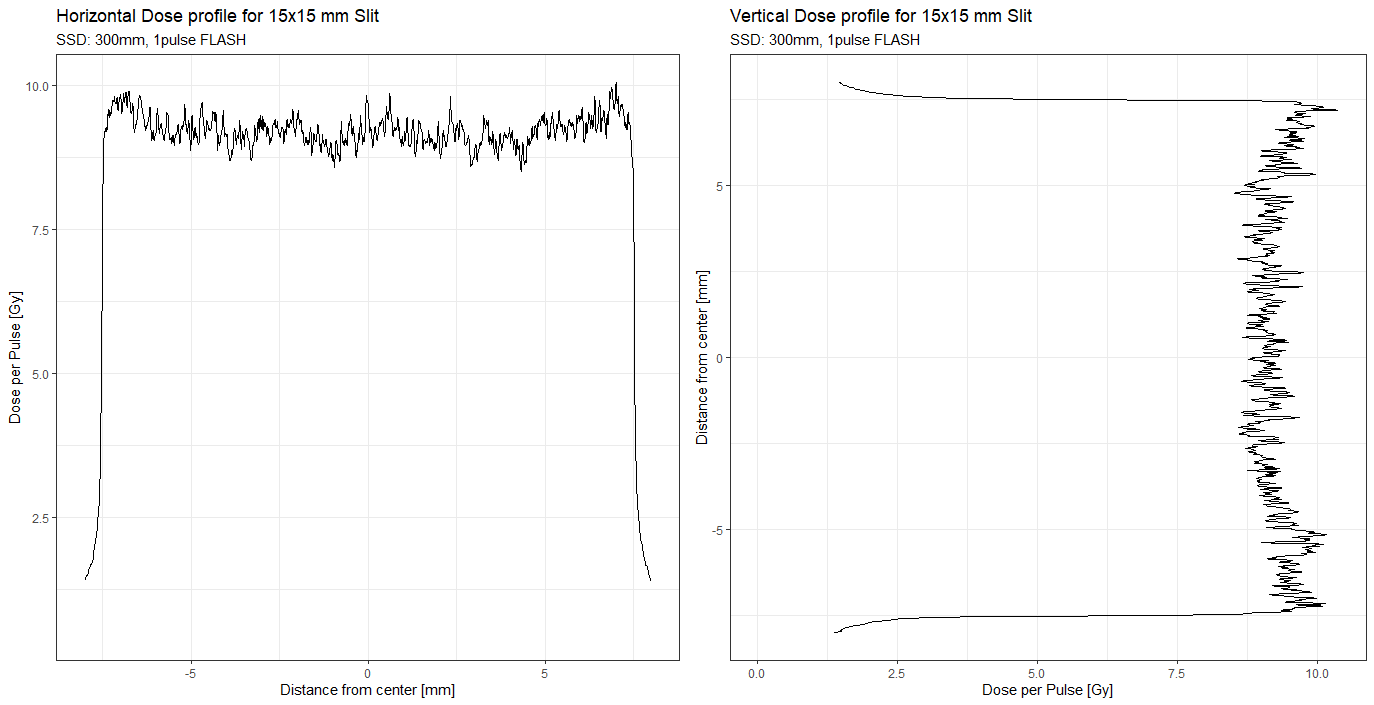 |
| ADR-volume histograms of beam for relevant structures | - | N/A |

Abbreviations: N/A = not applicable or not available.

**References**

1. Horst F, Brand M, Hans S, Karsch L, Lessmann E, Löck S, et al. Zebrafish Embryo Model of the FLASH Effect, In Regard to Böhlen et al. Int J Radiat Oncol [Internet] 2023 [cited 2023 Feb 28];115(4):1006–7. Available from: https://linkinghub.elsevier.com/retrieve/pii/S0360301622035295

2. Horst F, Bodenstein E, Brand M, Hans S, Karsch L, Lessmann E, et al. Dose and dose rate dependence of the tissue sparing effect at ultra-high dose rate studied for proton and electron beams using the zebrafish embryo model. Radiother Oncol [Internet] 2024 [cited 2024 Apr 24];194:110197. Available from: https://linkinghub.elsevier.com/retrieve/pii/S0167814024001191

3. Böhlen TT, Psoroulas S, Aylward JD, Beddar S, Douralis A, Delpon G, et al. Recording and reporting of ultra-high dose rate “FLASH” delivery for preclinical and clinical settings. Radiother Oncol [Internet] 2024 [cited 2024 Sep 9];110507. Available from: https://linkinghub.elsevier.com/retrieve/pii/S0167814024007771
